# How can the sustainable development goals improve the lives of people affected by conflict?

**DOI:** 10.2471/BLT.16.179622

**Published:** 2017-02-01

**Authors:** Emmanuel d’Harcourt, Ruwan Ratnayake, Anna Kim

**Affiliations:** a International Rescue Committee, 122 E 42nd Street, New York, New York 10168, United States of America.

One and a half billion people live in conflict-affected and fragile states. At the last estimate in 2012, 172 million people were directly affected by war, including refugees, internally displaced people and those who were affected but did not flee.^1^^,^^2^ Children are twice as likely to be malnourished and twice as likely to die by the age of five years in low-income countries affected by conflict compared with similar but stable countries. Their families are twice as likely to live without clean water.^3^ Conflict does more than short-term damage; it decimates a country’s infrastructure and impairs the social contract between the state and citizens. Food supplies are disrupted; health services collapse. Pregnant women and people who are ill do not receive the life-saving services they need.^4^ Less often measured are the long-term consequences of conflict on people’s mental health and social functioning.

People made vulnerable by conflict are being bypassed by global progress. The World Bank warned in 2011 that no low-income, conflict-affected country was on course to achieve any of the millennium development goals (MDGs).^3^ Indeed, four years later, of 55 conflict-affected and fragile states, 37 (67%) had met only two or fewer of the 15 MDG targets.^5^ The inequity is not simply about the differences between stable and unstable countries. Even within countries, conflict-affected areas fare worse than areas with less or no conflict. In the Democratic Republic of Congo, for example, under-five mortality in the conflict-affected South Kivu province is nearly double that of Kinshasa province.^6^

Despite this experience, the sustainable development goals (SDGs) for the year 2030 include barely more guidance on conflict than did the MDGs, which did not specifically mention conflict. SDG 16 explicitly recognizes the need to resolve conflict and mitigate its circumstances, but this intention does not translate into specific action points for other SDGs, such as SDG 3, which focuses on health. Unless we learn how to achieve the targets in conflict settings, the benefits of the SDGs will not reach many of the people who need them most.

The first question is whether the list of 17 SDGs and 169 targets should be adapted by each country. There is experience of simpler, more modest goals being associated with greater success. In Afghanistan, the government and its partners worked together to adapt the MDGs to meet their needs, setting more realistic interim targets and agreeing on objectives and approaches adapted to the country’s unique realities.^7^

Methods of measurement need to be realistic too. Widely used mechanisms to monitor progress in health at a national scale, such as the demographic and health surveys and the multiple indicator cluster surveys, sometimes leave out whole conflict-affected areas and routinely exclude internally displaced persons and refugees.^8^ There are alternatives, such as data collected by nongovernmental organizations that work in conflict zones. These data, which are collected to identify needs and monitor the progress of humanitarian interventions, are arguably underused for monitoring development goals.^8^ Population data are also scarce among displaced and disrupted communities, although humanitarian organizations commonly conduct and update small-scale censuses of difficult-to-access areas. Using these sources, while not a solution to the problem, could be a first step towards better monitoring.

We also need to be more realistic about the level of investment needed to effect even modest changes. Conflict-affected countries often have greater needs both in terms of capital costs to rebuild destroyed infrastructure and of recurring costs to operate in environments with transport and security challenges. Yet conflict-affected countries have received less investment than others. In 2012, for example, the Central African Republic received one-fifth the per-capita direct assistance for health that Malawi did.^9^ With less than 200 km of paved roads in South Sudan, a country larger than France, air transport is often the only option. Overall, levels of international humanitarian funding routinely face a large shortfall: in 2014, 7.2 out of 18 billion United States dollars of the United Nations (UN) coordinated appeal for humanitarian action worldwide went unfunded.^9^

Realistic goals, better measurement of progress and increased investment would help, but these will not be enough to make a difference without a fourth adaptation: the most difficult one. We need to change what we do and how we do it. Investment in scalable, cost-effective interventions, such as family planning, insecticide-impregnated bed nets and integrated community case management, have helped stable countries to make dramatic reductions in maternal and child mortality. Countries in crisis could and should benefit from similar public health interventions, but have often instead been served by short-term actions, such as provision of field hospitals and mobile clinics, which have higher costs, smaller scale and less potential for sustained impact. People already burdened by conflict receive aid that reaches fewer people, is more expensive and has a shorter impact than aid in non-conflict settings.

This does not mean implementing the same large-scale, long-term health programmes in fragile states as in stable low-income countries. Interventions should be effective and cost-effective and have wide coverage but they need to be adapted to the context of conflict. For example, integrated community case management for childhood diseases, traditionally considered a development intervention, has been adapted and scaled up in conflict-affected areas to increase access to care. In South Sudan, for example, the International Rescue Committee (IRC) supports over 2600 community health workers, many of them in the most intense areas of conflict such as Unity state. These community workers have continued to work even when conflict has shut down formal health structures. Tools were developed for illiterate community health workers, in a setting in which literacy rates are very low. Supply-chain adaptations, such as the use of boats and a network of transit villages to move materials, were developed to address problems of flooding and the lack of infrastructure.

Similarly, although family planning has nominally been included in the United Nations Population Fund (UNFPA) minimum initial service package for reproductive health, its family planning component has seldom been implemented in acute or chronic emergencies. In the last 10 years, a coalition of organizations with experience in reproductive health in conflict settings has demonstrated that modern and long-acting contraceptive methods can be provided at low cost and with high quality, and that there is demand for these, even in the most precarious and transient conditions.^10^^,^^11^ As a result, the full range of contraception methods are being provided by Save the Children, CARE, IRC and other agencies as a standard in places like the eastern Democratic Republic of Congo and northern Uganda, even in the midst of security upheavals. In other areas, such as Zaatari camp in Jordan, the United Nations High Commissioner for Refugees and its partner agencies are meeting the demand for modern contraception which existed before the Syrian refugee crisis and which continues.^12^

These approaches not only deliver proven, cost-effective interventions at much larger scale than most classic emergency interventions, but also help to mitigate the risks to humanitarian workers and health workers. Hospital workers in the Syrian Arab Republic and vaccine workers in Pakistan appear to have been deliberately targeted during recent conflicts. Attacks of this type are not new, but they are being increasingly documented. Community health workers are less visible than facility workers, and can provide vital services with less danger to themselves in settings where formal health workers are deliberately targeted. They consequently have more options for continuing services in conflict.

Such new approaches to aid in conflicts will not happen without a new understanding of coordination that lasts beyond the acute phase of conflict, and that includes both acute and long-term actors. The cluster system, a UN-led, country-specific coordination system for acute emergencies, has helped to bring better geographical distribution of aid and better communication between agencies. What is needed, however, is coordination not just among acute responders but also among health actors with complementary mandates and spheres of action. Agencies with expertise in specific interventions, such as UNFPA or Planned Parenthood affiliates in family planning, need to support acute responders, such as Médecins Sans Frontières. Agencies just arriving into a conflict area need to collaborate with agencies within a country who can contribute greater knowledge of the local context and assets such as networks of community health workers. Establishing more effective public health responses will also require better coordination with governments ‒ a challenging task in many conflict settings, but an important one. Last but not least, an approach combining large-scale public health interventions with responses in conflict settings will require coordination across different kinds of donors and sometimes different teams within the same agency who may not be used to coordinating among themselves.

As the world embarks on another 15-year enterprise in global aid planning, implementation and tracking, we owe it to populations affected by conflict – who are some of the most vulnerable people on earth – to apply public health principles to deliver better aid. Changing the politics that drive conflict is beyond the sphere of the global health community. However, it is well within our power to maintain our high standards of effectiveness and common sense regarding cost‒effectiveness, and to coordinate better between global health actors. With a smarter, more adapted, more ambitious approach to assistance in conflict settings, we have an opportunity to make the SDGs more effective and more equitable than previous development goals.
